# Influence of different frequencies of transcutaneous electrical nerve stimulation on the threshold and pain intensity in young subjects

**DOI:** 10.1590/S1679-45082014AO3092

**Published:** 2014

**Authors:** Adriana de Oliveira Gomes, Ana Caroline Silvestre, Cristina Ferreira da Silva, Mariany Ribeiro Gomes, Maria Lúcia Bonfleur, Gladson Ricardo Flor Bertolini

**Affiliations:** 1 Universidade Estadual do Oeste do Paraná, Cascavel, PR, Brazil.

**Keywords:** Pain, Pain measurement, Electric stimulation, Cold temperature, Pressure

## Abstract

**Objective:**

To investigate the effects of different transcutaneous electrical nerve stimulation frequencies in nociception front of a pressure pain threshold and cold in healthy individuals.

**Methods:**

Twenty healthy subjects were divided into four groups, all of which have gone through all forms of electrical stimulation at different weeks.

**Assessments were:**

pre and post-therapy, 20 and 60 minutes after stimulation. To evaluate the pressure pain threshold, an algometer was used with one tapered tip, pressing the hypothenar region until voluntary report the word “pain”. Cold pain intensity was assessed by immersion in water at 5°C for 30 seconds; at the end, the subject was asked to quantify the pain intensity on a Visual Analog Scale for Pain. For electrical stimulation, two electrodes were used near the elbow, for 20 minutes, with an intensity strong, but not painful. The frequency was in accordance with the group: 0Hz (placebo); 7Hz; 100Hz; and 255Hz.

**Results:**

Both for the assessment of pressure pain threshold as the cold pain intensity, there was no significant difference (p>0.05).

**Conclusion:**

We conclude that the use of transcutaneous electrical nerve stimulation on dermatomes C6 to C8 produced no significant change in pressure pain threshold or cold discomfort.

## INTRODUCTION

Pain is a multidimensional phenomenon with sensitive, emotional, and cognitive components, described by the International Association for the Study of Pain as an unpleasant, emotional, and sensorial experience, linked or not to organic damage or described by the patient in such terms.^(1)^ Nervous electrical stimulation (TENS - transcutaneous electrical nerve stimulation) is a noninvasive treatment used in physical therapy practice to promote analgesia^(2)^, which has been increasing used due to its easy application and for requiring less administration of drugs, thus promoting the patient’s well-being and reduction of costs with treatment.^(3)^

TENS is a low intensity alternated current that produces electrical impulses of various frequencies, and is effective in treating musculoskeletal disorders, as it influences and modulates nerve conduction processes of pain. It acts on peripheral mechanoceptors in which the stimulus is conducted by fibers Aβ, with long diameter, to the set of interneuron, which act in inhibiting retransmission of pain stimuli conducted by fibers Aδ and C, both with narrow diameters, closing the compartment of pain.^(4, 5)^ Additionally, TENS may also produce the release of serotonin, reduce the action of aspartate and glutamate on the spine,^(6)^ and at low frequencies, there is participate of endogenous opioids.^(2, 7)^

In humans, some models of induced pain are used with the intent of evaluating the use of analgesic modalities such as electrostimulation. One of them is the model of pain induced by cold, a simple method that involves a minimal risk of tissue lesion and in which pain ceases upon removal of the stimulus. During this test, a painful sensation is generated by the temperature receptors that start to send stimuli to a possible tissue damage site by peripheral routes (fibers C and fibers Aδ) and central routes (spinothalamic and spinoreticular), resulting in the sensation of pain induced by the cold.^(8)^

The pressure algometer is the other instrument which is useful and reliable for determining pressure pain threshold. It may be placed along a reference point, and the pressure is increased slowly. The quantity of pressure is usually recorded as pain threshold to pressure, which is the level at which subjects report feeling discomfort.^(9)^

Despite TENS being amply used, there are still conflicting results as to the analgesic effects produced by electrostimulation and its parameters, such as frequency.^(10)^ Therefore, studies that cover such variables in experiments that involve the threshold and intensity of pain in humans are yet needed.

## OBJECTIVE

To investigate the effects of different frequencies of TENS in nociception regarding pressure pain stimulus and cold in healthy individuals.

## METHODS

Twenty healthy individuals participated in the study (14 males), aged 22.2±3.07 years, weight 74.95±19.69kg, height 1.71±0.08m, and body mass index (BMI) 25.12±4.95. The size of the sample was based on prior laboratory studies about the use of the pressure algometer on the hypothenar region, with a standard deviation of 0.37, difference to be detected at 0.3, with a significance level of 5%, and test power of 80%.

After explanation as to the objectives and procedures of the study, the volunteers were submitted to triage for data recording and to identify possible exclusion factors. As inclusion criteria, the volunteers were to be available to participate in assessments and tests on the predetermined days and times and show sensitivity integrity of dermatomes C6 and C8. The exclusion criteria were contraindication for the use of any type of electrostimulation; individuals who used pacemakers or any important metallic implant; the presence of a febrile state, neoplasm, tuberculosis, cognitive or sensorial deficit, suspected or confirmed diagnosis of deep vein thrombosis; and those who did not show up for data collection. After eligibility for the study was confirmed, the volunteers signed the Informed Consent Form.

Those evaluated were randomly allocated by drawing of names from an opaque envelope, to four subgroups with five individuals each (Groups A, B, C, and D). Among the subgroups, the volunteers received the same frequency of stimulation, with a change in frequency as per the week of the experiment (Table 1). The 20 volunteers underwent the different forms of electrostimulation: TENS 1 (GT1 – 0Hz), TENS 2 (GT2 – 7Hz), TENS 3 (GT3 – 100Hz), and TENS 4 (GT4 – 255Hz), with evaluations divided into four moments: pre-application, post-application, 20 minutes, and 60 minutes after electrostimulation.

**Table 1:** Demonstration of the sequence and weekly intervention for the volunteers, as per group and subgroup (different frequencies)

Group	Week			
	1 (Hz)	2 (Hz)	3 (Hz)	4 (Hz)
A	0	7	100	255
B	7	100	255	0
C	100	255	0	7
D	255	0	7	100

### Evaluation of pain by stimulation of mechanoreceptors

Before beginning the first evaluation, the volunteers were instructed to immerge the upper dominant limb, up to the elbow, into a Kc-700 Turbo Hidro Kroman^®^ water swirling tank for 5 minutes, in warm water at 38°C, in order to produce thermal equilibrium among the participants. Next, to evaluate the pain by pressure threshold, a Kratos^®^ brand algometer with capacity to produce up to 50Kgf of pressure was used. The volunteers were explained that the pain would be evaluated by means of a pressure stimulation technique, and the individual should report the moment he/she felt it.^(11)^ Assessment with the pressure algometer was made by only one evaluator, and the device was used with a metallic tapered-end stem in the hypothenar region, 3cm from the wrist fold, with gradual vertical pressure, until the volunteer reported the word “pain”. After measuring, the Kgf force necessary to produce the painful stimulus in each individual was noted (AV1).

### Evaluation of pain by stimulation of thermoreceptors

The intensity of pain by cold was evaluated by means of immersion up to the level of the cubital fossa in a plastic recipient with 34cm diameter and 36cm height, with water at 5°C, during 30 seconds. The water temperature was constantly verified with a thermometer (Incoterm^®^). Next, the participant was told to remove the limb from the cold water and to mark the intensity of pain on a Visual Analog Scale (VAS) of Pain.^(11)^

### TENS application protocol

**Figure 1:**
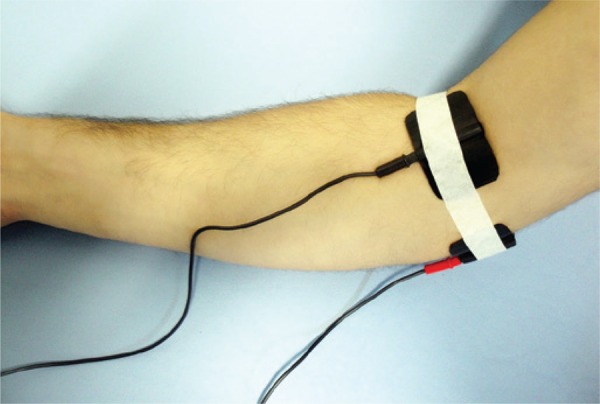
Positioning of electrodes on the superficial region of the median and ulnar nerves, near the elbow joint

After the first evaluation, the individual was taken to a room when another researcher applied TENS (Bioset^®^). Two 8cm^2 ^non-adhesive electrodes (silicone rubber with carbon) with a conductive medium (water-based hypoallergenic gel) were used. Initially, the site was cleaned with 70% alcohol and sterile cotton; next, the electrodes were positioned closed to the elbow and affixed with tape. One electrode was placed between the medial epicondyle and the olecranon (ulnar nerve sulcus), and the other, medial to the insertion of the brachial biceps (median nerve region) during 20 minutes, with an intensity described as strong, but not painful (Figure 1). Duration of the phase used was 250µs, and the frequency was according to the group: GT1 – device turned on, but with no current passing through (0Hz); GT2 – low frequency (7Hz); GT3 – high frequency (100Hz); GT4 – maximal frequency available on the equipment (255Hz).

For GT1, the volunteers were induced to believe they were being submitted to an electrostimulation below the sensitivity threshold, in which they did would not feel paresthesia. Each TENS group received an application of a determined frequency a week, and at the end of 4 weeks, the four groups were submitted to the four types of frequency of the study (Table 1).

After TENS application, the individual returned to the previous room for reevaluations of pressure and pain by cold.

### Statistical analysis

Analysis of normality was performed by means of the Kolmogorov-Smirnov test, and then, data were analyzed with variance analyses of repeated measurements (ANOVA), with a 5% significance level.

The evaluations were made at the *Clínica de Fisioterapia da Universidade Estadual do Oeste do Paraná *(UNIOESTE), Cascavel campus, between the months of August and September 2013. The experimental procedures were approved by the Ethics in Research Committee of the institution under number 115/2013-CEP, based on Resolution 196/96 of the National Council of Health.

## RESULTS

There was no significant difference either for the evaluation of pain threshold due to pressure or the intensity of cold (p>0.05) (Table 2).

**Table 2:** Mean of the values found for the threshold of pain by pressure and intensity of pain by cold for the different frequencies for application of transcutaneous nervous stimulation at the various moments of evaluation

		AV1	AV2	AV3	AV4
Threshold of pain due to pressure (kgf)	GT1	0.751±0.342	0.697±0.241	0.681±0.279	0.765±0.388
	GT2	0.806±0.299	0.719±0.268	0.707±0.278	0.566±0.236
	GT3	0.744±0.323	0.777±0.425	0.789±0.425	0.671±0.334
	GT4	0.688±0.268	0.735±0.326	0.689±0.253	0.705±0.223
Intensity of pain by cold (cm)	GT1	3.39±2.14	3.06±1.38	3.35±2.03	3.49±2.04
	GT2	3.69±2.47	3.88±2.28	3.72±1.95	3.52±2.13
	GT3	3.36±2.07	4.01±2.02	3.61±1.65	3.36±2.01
		3.70±2.10	3.47±1.96	3.14±1.58	3.49±2.06

There were no significant differences (p>0.05 – ANOVA with repeated measurements).

AV1: first evaluation; AV2: second evaluation; AV3: third evaluation; AV4: fourth evaluation; GT1: TENS1 Group (0Hz); GT2: TENS2 Group (7Hz); GT3: TENS3 Group (100Hz); GT4: TENS4 Group (255Hz).

## DISCUSSION

TENS is credited with analgesia through various mechanisms,^(2, 4)-7)^ and the most probably mechanism evaluated in this study was the blockage or the increase in threshold of nervous fiber depolarization. Since the volunteers were healthy, and the pain stimulus was induced, we evaluated a possible segmental analgesia located in the dermatome resulting from the interference of the pain message, and conventional TENS may, therefore, be responsible for this effect, since it interferes in the transmission of pain sensations to the supraspinal levels.^(7, 12)^

Previous studies have shown that low frequency TENS may affect analgesia via the release of endogenous opioids.^(2, 7)^ The use with frequencies close to 100Hz may produce effects of through the release of serotonin,^(6)^ but higher frequencies, such as 255Hz found in the equipment, are not commonly evaluated. Although TENS is effective in treating acute pain, in this study this fact was not observed, regardless of the frequency used: neither 7Hz, 100Hz, nor 255Hz was effective in increasing the pain threshold induced by pressure in the hypothenar region in our evaluations, which is in agreement with the results obtained by Schulz et al.^(4)^ on the action of TENS in the form of Burst on the threshold of pain induced by pressure. This study also did not display differences of pain caused by cold (superficial), similar to what was found by Morimoto et al.^(8)^ when stimulating with 80Hz or 4Hz. On the other hand, Montenegro et al.,^(1)^ when stimulating with TENS at acupuncture points, did not observe effects on the intensity of pain, but there was an increase in pain threshold to cold, a fact also observed by Santos et al.^(13)^

According to Claydon et al.,^(10)^ the results of TENS are conflicting, but there is moderate evidence for the use of this current at high intensity. Whereas for conventional TENS, there is strong evidence of effects for pain caused by pressure and of the absence of effects on ischemic pain. Nevertheless, the same authors,^(14)^ using an equipment with frequency varying every 3 seconds (4Hz/110Hz), did not observe any difference between the groups stimulated and the placebo and control groups. This fact was also observed in the present study, since there was no significant difference relative to the threshold of pain by pressure in the hypothenar region, both in the intra-comparison and among the groups. Lazarou et al.^(15)^ observed that low frequencies (2Hz) at high intensities (pain threshold) showed hypoalgesic effects, but there was no response when the intensity was low (strong, but comfortable). This is different from what was observed by Farias et al.,^(16)^ who used an adipometer to evaluate light pain in healthy volunteers, and reported analgesic levels of TENS (100Hz) at sensitive limits. In agreement with Moran et al.,^(17)^ who despite observing an intensity-dependent effect had hypoalgesic effects starting at the sensitive threshold.

In a prior study, Claydon et al.^(18)^ observed that segment and extra-segment stimulations in healthy volunteers showed hypoalgesic effects on the threshold of pain by pressure for groups in which high intensities were used (pain tolerance threshold), but were not able to obtain results different from those obtained for the placebo when the intensity was low (strong, but comfortable, similar to that used in the present study). The authors point out that it is probable that, with high intensities, fibers Aδ and C are stimulated, producing local descending inhibitory mechanisms to the area of stimulation and diffuse damaging inhibitory control, and perhaps the need for high intensities to effectively initiate these mechanisms, that is, uncomfortable stimulation. It is believed that with the stimulation parameters used in the present study, no alterations occurred in the action of pain receptors or alterations of the descending analgesic routes. Since the intensity used did not reach levels of discomfort, it is believed that such explanations may be valid for the results found in this study, since it is a sample without any underlying pain-producing disease, as was observed by Morgan and Santos^(5)^ in patients with osteoarthrosis of the knee, when observing analgesic effects of TENS on a sensitive level. We point out that, despite the use of comfortable intensities, this was increased at all the moments in which the volunteers reported accommodation of stimulation.^(19)^

In the study performed by Palmeira et al.,^(20)^ significant differences were observed between the genders relative to the tolerance threshold and discomfort to pain, in which men reported greater resistance to pain than women, while women reported greater disposition. Of all the types of experimental pain, pressure pain, in particular, seems to be the most sensitive to gender differences. In the present study, there were both male and female volunteers, making the cross-over study design interesting when the objective was to avoid biases related to gender. Additionally, we highlight the importance of an absolute control group, that is, that receives no electrostimulation and is aware of this,^(15, 21)^ which was one of the limitations of this study.

The time of 20 minutes of electrostimulation used in this study was demonstrated by Liebano et al.^(21)^ as being capable of producing analgesia to pressure, both at high (100Hz) and low frequencies (4Hz) at the maximum intensity tolerable. A care taken in the present study was standardization of the initial temperature, before the first evaluation. In a study conducted by Morimoto et al.,^(8)^ before each assessment, the member evaluated was immersed in warm water so that the temperature was uniform in each one of the evaluations. Such care not taken in the present study may have caused alterations in the thresholds of pain detected.

In this way, besides the difference in gender, we highlight as limitations of the study the non-stabilization of the temperature prior to each evaluation and the lack of an absolute control group, i.e., individuals who did not go through any type of electrical stimulation, which did not happen here since it was a cross-over study with all the volunteers receiving all the forms of electrostimulation.

For future studies, attention is suggested for these issues, besides protocols with treatment times superior to 20 minutes, and, primarily, the use of greater intensities close to the threshold of pain.

## CONCLUSION

The use of transcutaneous electrical nervous stimulation on dermatomes C6 to C8, with application of 0, 7, 100, and 255Hz, produced no significant alterations in the threshold of pain by pressure or discomfort caused by cold.
