# Supplementary material for: Influence of different frequencies of transcutaneous electrical nerve stimulation on the threshold and pain intensity in young subjects
Source: Einstein (Sao Paulo). 2014 Jul-Sep;12(3):318–22. doi: 10.1590/S1679-45082014AO3092 (PMC4872943; doi:10.1590/S1679-45082014AO3092)
Supplement: Translation

# Translation: As influências de diferentes frequências da estimulação elétrica nervosa transcutânea no limiar e intensidade de dor em indivíduos jovens

**Authors:** Adriana de Oliveira Gomes, Ana Caroline Silvestre, Cristina Ferreira da Silva, Mariany Ribeiro Gomes, Maria Lúcia Bonfleur, Gladson Ricardo Flor Bertolini

**Affiliations:** 1 Universidade Estadual do Oeste do Paraná, Cascavel, PR, Brasil.

**Keywords:** Dor, Medição da dor, Estimulação elétrica, Temperatura baixa, Pressão

## Abstract

**Objetivo:**

Investigar os efeitos de diferentes frequências da estimulação elétrica nervosa transcutânea na nocicepção, frente a um estímulo doloroso pressórico e ao frio, em indivíduos saudáveis.

**Métodos:**

Participaram 20 indivíduos saudáveis, divididos em 4 grupos, sendo que todos passaram por todas as formas de eletroestimulação, em semanas diferentes. As avaliações ocorreram nos seguintes períodos: pré-aplicação, pós-aplicação, 20 e 60 minutos após a eletroestimulação. Para avaliar o limiar de dor à pressão, foi utilizado um algômetro com ponta afilada, pressionando na região hipotenar, até o voluntário relatar a palavra “dor”. A intensidade de dor ao frio foi avaliada por meio de imersão em água a 5°C, durante 30 segundos; ao final, pediu-se para que o indivíduo quantificasse a intensidade álgica em uma Escala Visual Analógica de Dor. Para a eletroestimulação, foram utilizados dois eletrodos próximos ao cotovelo, durante 20 minutos, com intensidade referida como forte, porém não dolorosa. A frequência esteve de acordo com o grupo: 0Hz (placebo); 7Hz; 100Hz; e 255Hz.

**Resultados:**

Tanto para a avaliação do limiar de dor à pressão quanto da intensidade ao frio, não houve diferença significativa (p>0,05).

**Conclusão:**

O uso da estimulação elétrica nervosa transcutânea, sobre os dermátomos de C6 a C8, não produziu alteração significativa no limiar de dor à pressão e nem no desconforto ao frio.

## INTRODUÇÃO

A dor é um fenômeno multidimensional com componentes sensitivos, emocionais e cognitivos, sendo descrita, segundo a Associação Internacional para o Estudo da Dor, como uma experiência desagradável, emocional e sensorial, ligada ou não a um dano orgânico, ou descrito pelo paciente em tais termos.^(1)^ A estimulação elétrica nervosa transcutânea (TENS, sigla do inglês *transcutaneous electrical nerve stimulation*) é um tratamento não invasivo usado na prática fisioterapêutica para promover analgesia^(2)^ que tem sido cada vez mais utilizada por sua fácil aplicação e por propiciar menor necessidade de administração de fármacos, promovendo, assim, o bem-estar do paciente e a redução de custos com o tratamento.^(3)^

A TENS é uma corrente alternada, de baixa intensidade, que produz impulsos elétricos de várias frequências, sendo eficaz no tratamento de desordens musculoesqueléticas, por influenciar e modular processos de neurocondução da dor. Atua sobre os mecanoceptores periféricos, sendo o estímulo conduzido pelas fibras Aβ, de diâmetro largo, até o conjunto de interneurônios, que atua na inibição da retransmissão dos estímulos dolorosos conduzidos pelas fibras Aδ e C, ambas de diâmetros estreitos, fechando a comporta da dor.^(4, 5)^ Além dessa, a TENS também pode produzir liberação de serotonina, reduzir a ação do aspartato e glutamato sobre a medula,^(6)^ e, em baixas frequências, há participação de opioides endógenos.^(2, 7)^

Em humanos, alguns modelos de dor induzida são utilizados, visando avaliar o uso de modalidades analgésicas, como a eletroestimulação. Um deles é o modelo de dor induzida pelo frio, um método simples que envolve um risco mínimo de lesão tecidual e no qual a dor cessa após a remoção do estímulo. Durante esse teste, uma sensação dolorosa é gerada pelos receptores de temperatura, que começam a enviar estímulos de um possível dano tecidual pelas vias periférica (fibras C e Aδ**)** e central (espinotalâmica e espinoreticular), resultando na sensação de dor induzida pelo frio.^(8)^

O algômetro de pressão é outro instrumento útil e de uso confiável para determinar limiar da dor de pressão. Pode ser colocado ao longo de um ponto de referência, e a pressão é aumentada lentamente. A quantidade de pressão é normalmente registrada como limiar da dor à pressão, o qual é o nível em que os sujeitos relatam desconforto.^(9)^

Apesar da TENS ser amplamente utilizada, existem resultados conflitantes com relação aos efeitos analgésicos produzidos pela eletroestimulação e os parâmetros do mesmo, como, por exemplo, a frequência.^(10)^ São necessários, desse modo, estudos que abordem tais variáveis em experimentos envolvendo limiar e intensidade de dor em humanos.

## OBJETIVO

Investigar os efeitos de diferentes frequências da TENS na nocicepção, frente a um estímulo doloroso pressórico e ao frio, em indivíduos saudáveis.

## MÉTODOS

Participaram do estudo 20 indivíduos saudáveis (14 do sexo masculino), com idades de 22,2±3,07 anos, peso de 74,95±19,69kg, altura de 1,71±0,08m e índice de massa corporal (IMC) de 25,12±4,95. O tamanho da amostra baseou-se em estudos prévios do laboratório sobre o uso do algômetro de pressão na região hipotenar, com desvio-padrão de 0,37, diferença a ser detectada 0,3, nível de significância de 5% e poder de teste de 80%.

Após o esclarecimento acerca dos objetivos e dos procedimentos do estudo, os voluntários foram submetidos à triagem para o registro de dados e para identificação de possíveis fatores de exclusão. Como critérios de inclusão, os voluntários deveriam ter disponibilidade para participar das avaliações e dos testes nos dias e horários predeterminados e apresentar integridade sensitiva dos dermátomos C6 e C8. Os critérios de exclusão foram contraindicação para o uso de qualquer tipo de eletroestimulação; indivíduos que fizessem o uso de marca-passo ou de qualquer implante metálico; presença de estado febril, neoplasia, tuberculose, défice cognitivo ou sensorial, suspeita ou diagnóstico de trombose venosa profunda; e os que não compareceram para a coleta de dados. Depois de constatada a elegibilidade para o estudo, os voluntários assinaram o Termo de Consentimento Livre e Esclarecido.

Os avaliados foram alocados, de maneira aleatória, por sorteio dos nomes em envelope opaco, em quatro subgrupos de cinco indivíduos (Grupos A, B, C e D). Dentro dos subgrupos, os voluntários recebiam a mesma frequência de estimulação, havendo mudança de frequência de acordo com a semana do experimento (Tabela 1). Os 20 voluntários passaram pelas diferentes formas de eletroestimulação: TENS 1 (GT1 – 0Hz), TENS 2 (GT2 – 7Hz), TENS 3 (GT3 – 100Hz) e TENS 4 (GT4 – 255Hz), com avaliações divididas em quatro momentos: pré-aplicação, pós-aplicação, 20 minutos e 60 minutos após a eletroestimulação.

**Tabela 1:** Demonstração da sequência e intervenção semanal para os voluntários, de acordo com o grupo e subgrupo (diferentes frequências)

Grupo	Semana			
	1 (Hz)	2 (Hz)	3 (Hz)	4 (Hz)
A	0	7	100	255
B	7	100	255	0
C	100	255	0	7
D	255	0	7	100

### Avaliação da dor pelo estímulo dos mecanoceptores

Antes do início da primeira avaliação, os voluntários foram orientados a imergir o membro superior dominante, até o cotovelo, em um turbilhão Kc-700 Turbo Hidro Kroman^®^, por 5 minutos, em água morna a 38°C, visando produzir equilíbrio térmico entre os participantes. Em seguida, utilizou-se, para avaliar o limiar de dor à pressão, um algômetro da marca Kratos^®^, com capacidade de produzir pressão em até 50Kgf. Foi explicado aos voluntários que a dor seria avaliada por meio de uma técnica de estimulação por pressão, e o indivíduo deveria relatar o momento em que começava a senti-la.^(11)^ A avaliação com o algômetro de pressão foi feita por apenas um avaliador, e o aparelho foi utilizado com uma haste metálica com ponta afilada, pressionando na região hipotenar, 3cm a partir da prega proximal do punho, com pressão gradual, vertical, até o voluntário relatar a palavra “dor”. Após a mensuração, foi anotada a força em Kgf necessária para ocorrer o estímulo doloroso em cada indivíduo (AV1).

### Avaliação da dor pelo estímulo dos termoceptores

A intensidade de dor ao frio foi avaliada por meio de imersão até o nível da fossa cubital, em um recipiente plástico com diâmetro de 34cm e 36cm de altura, com água a 5°C, durante 30 segundos. A temperatura da água era constantemente verificada com termômetro (Incoterm^®^). Passado esse tempo, pediu-se para que o indivíduo retirasse o membro da água gelada e marcasse a intensidade álgica em uma Escala Visual Analógica de Dor (EVAD).^(11)^

### Protocolo de aplicação da TENS

Após a primeira avaliação, o indivíduo foi conduzido a uma sala, onde outro pesquisador aplicou a TENS (Bioset^®^). Foram utilizados dois eletrodos não adesivos (borracha siliconada com carbono) de 8cm^2^ com meio condutivo (gel hipoalergênico à base de água). Inicialmente, foi feita a higienização do local com álcool 70% e algodão estéril; em seguida, os eletrodos foram posicionados próximos ao cotovelo e fixados com fita crepe. Um eletrodo foi disposto entre o epicôndilo medial e olécrano (sulco do nervo ulnar), e o outro medial à inserção do bíceps braquial (região do nervo mediano), durante 20 minutos, com intensidade referida como forte, porém não dolorosa (Figura 1). A duração de fase utilizada foi de 250µs, e a frequência foi de acordo com o grupo: GT1 – aparelho ligado, porém sem passagem de corrente (0Hz); GT2 – baixa frequência (7Hz); GT3 – alta frequência (100Hz); GT4 – a máxima frequência disponível no equipamento (255Hz).

**Figura 1:** Posicionamento dos eletrodos na região de superficialidade dos nervos mediano e ulnar, próximos à articulação do cotovelo

Para GT1, os voluntários foram induzidos a acreditar que estava sendo submetido a um tratamento de eletroestimulação abaixo do limiar sensitivo, no qual os voluntários não sentiriam parestesia. Cada grupo TENS recebeu uma aplicação de determinada frequência por semana, sendo que, ao final das 4 semanas, os quatro grupos foram submetidos aos quatro tipos de frequências do estudo (Tabela 1).

Após a aplicação da TENS, o indivíduo regressou à sala anterior, para as reavaliações de pressão e dor ao frio.

### Análise estatística

A análise da normalidade foi realizada por meio do teste de Kolmogorov-Smirnov, em seguida, os dados foram analisados com análises de variância (ANOVA) medidas repetidas, com nível de significância de 5%.

As avaliações foram realizadas na Clínica de Fisioterapia da Universidade Estadual do Oeste do Paraná (UNIOESTE), campus Cascavel, entre os meses de agosto e setembro de 2013. Os procedimentos experimentais foram aprovados pelo Comitê de Ética e Pesquisa da instituição sob número 115/2013-CEP, com base na resolução 196/96 do Conselho Nacional de Saúde.

## RESULTADOS

Tanto para a avaliação do limiar de dor à pressão quanto da intensidade ao frio, não houve diferença significativa (p>0,05) (Tabela 2).

**Tabela 2:** Média dos valores encontrados para o limiar de dor à pressão e intensidade de dor ao frio, para as diferentes frequências de aplicação da estimulação elétrica nervosa transcutânea, nos diferentes momentos de avaliação

		AV1	AV2	AV3	AV4
Limiar de dor à pressão (kgf)	GT1	0,751±0,342	0,697±0,241	0,681±0,279	0,765±0,388
	GT2	0,806±0,299	0,719±0,268	0,707±0,278	0,566±0,236
	GT3	0,744±0,323	0,777±0,425	0,789±0,425	0,671±0,334
	GT4	0,688±0,268	0,735±0,326	0,689±0,253	0,705±0,223
Intensidade de dor ao frio (cm)	GT1	3,39±2,14	3,06±1,38	3,35±2,03	3,49±2,04
	GT2	3,69±2,47	3,88±2,28	3,72±1,95	3,52±2,13
	GT3	3,36±2,07	4,01±2,02	3,61±1,65	3,36±2,01
	GT4	3,70±2,10	3,47±1,96	3,14±1,58	3,49±2,06

Não houve diferenças significativas (p>0,05 – ANOVA medidas repetidas).

AV1: primeira avaliação; AV2: segunda avaliação; AV3: terceira avaliação; AV4: quarta avaliação; GT1: Grupo TENS1 (0Hz); GT2: Grupo TENS2 (7Hz); GT3: Grupo TENS3 (100Hz); GT4: Grupo TENS4 (255Hz).

## DISCUSSÃO

À TENS é creditada analgesia por diversos mecanismos,^(2, 4)-7)^ sendo o provável mecanismo avaliado neste estudo o bloqueio ou o aumento do limiar de despolarização de fibras nervosas. Visto que eram voluntários saudáveis e que o estímulo de dor foi induzido, avaliou-se uma possível analgesia segmentar localizada no dermátomo decorrente da interferência da mensagem dolorosa.^(8)^ Essa analgesia também pode ser interpretada como um aumento no limiar da dor, e a TENS convencional pode ser responsável por esse efeito, uma vez que interfere na transmissão das sensações dolorosas para os níveis supraespinais.^(7, 12)^

Trabalhos anteriores mostram que a TENS de baixa frequência pode atingir analgesia via liberação de opioides endógenos.^(2, 7)^ O uso em frequências próximas a 100Hz pode produzir efeitos via liberação de serotonina,^(6)^ mas frequências mais altas, como 255Hz, encontradas nos equipamentos, não são comumente avaliadas. Embora a TENS seja eficaz no tratamento da dor aguda, neste estudo esse fato não foi observado, independentemente da frequência utilizada; 7Hz,100Hz ou 255Hz, nenhuma delas foi eficaz em aumentar o limiar de dor induzida por pressão na região hipotenar nas avaliações, estando de acordo com os resultados obtidos por Schulz et al.^(4)^ sobre a ação da TENS na forma *Burst* sobre o limiar de dor induzido por pressão. O presente estudo também não apresentou diferenças na intensidade de dor ao frio (superficial), semelhantemente ao que foi encontrado por Morimoto et al.^(8)^ ao estimular com 80Hz ou 4Hz. Já Montenegro et al.,^(1)^ ao estimularem com TENS em pontos de acupuntura, não observaram efeito sobre a intensidade da dor, mas houve aumento do limiar de dor ao frio, fato também observado por Santos et al.^(13)^

Segundo Claydon et al.,^(10)^ os resultados da TENS são conflitantes, mas há moderada evidência para o uso dessa corrente em alta intensidade. Já para o TENS convencional, existem fortes evidências de efeitos para a dor à pressão e também de ausência de efeitos na dor isquêmica. No entanto, os mesmos autores,^(14)^ utilizando um equipamento com frequência variada a cada 3 segundos (4Hz/110Hz), não observaram diferenças dos grupos estimulados com os grupos placebo e controle. Tal fato também foi observado no presente estudo, visto que não houve diferença significativa em relação ao limiar de dor à pressão, na região hipotenar, tanto na comparação intra, quanto entre os grupos. Lazarou et al.^(15)^ observaram que baixas frequências (2Hz) em intensidades altas (umbral de dor) apresentaram efeitos hipoalgésicos, mas não houve resposta quando a intensidade foi baixa (forte, mas confortável). Diferente do observado por Farias et al.,^(16)^ que, utilizando um adipômetro para avaliar dor leve em voluntárias saudáveis, relataram níveis analgésicos da TENS (100Hz) em limites sensitivos. Concordante com Moran et al.,^(17)^ que, apesar de observarem um efeito intensidade-dependente, tiveram efeitos hipoalgésicos, a partir do limiar sensitivo.

Em estudo prévio, Claydon et al.^(18)^ observaram que as estimulações segmental e extrassegmental em voluntários saudáveis apresentaram efeitos hipoalgésicos no limiar de dor à pressão para grupos em que foram utilizadas altas intensidades (umbral de tolerância da dor), mas não conseguiram obter resultados diferentes dos obtidos para o placebo quando a intensidade foi baixa (forte, mas confortável, semelhante ao utilizado no presente estudo). Os autores ressaltaram que é provável que, com altas intensidade, estimulam-se fibras Aδ e C, produzindo mecanismos inibitórios descendentes locais para a área de estimulação e controle inibitório nocivo difuso, sendo plausível a necessidade de altas intensidades para efetivamente iniciar esses mecanismos, ou seja, estimulação desconfortável. Acredita-se que, com os parâmetros de estimulação utilizados no presente estudo, não ocorram alterações na ação dos receptores de dor, e nem mesmo alteração das vias analgésicas descendentes. Uma vez que a intensidade utilizada não atingiu níveis de desconforto, crê-se que tais explicações podem ser válidas para os resultados aqui encontrados, por se tratar de uma amostra sem doenças de base que produzam dor, como o observado por Morgan e Santos^(5)^, em pacientes com osteoartose de joelho, ao observaram efeitos analgésicos do TENS em nível sensitivo. Salienta-se que, apesar do uso de intensidades confortáveis, a mesma era aumentada em todos os momentos que os voluntários relatavam acomodação da estimulação.^(19)^

No estudo de Palmeira et al.,^(20)^ observaram-se diferenças significativas entre os sexos em relação ao limiar, à tolerância e ao desconforto à dor, sendo que os homens relataram maior resistência à dor do que as mulheres, enquanto as mulheres relataram maior disposição. De todos os tipos de dor experimental, a dor à pressão, em particular, parece ser mais sensível às diferenças sexuais. No presente estudo, existiam tanto voluntários homens quanto mulheres, tornando a forma de estudo cruzado interessante, ao se objetivar evitar vieses relacionados ao sexo. Ainda, salienta-se que a importância de um grupo controle absoluto, ou seja, que não receba a eletroestimulação e que esteja ciente disso,^(15, 21)^ sendo esta uma das limitações do presente estudo.

O tempo de 20 minutos de eletroestimulação utilizado neste estudo foi demonstrado por Liebano et al.^(21)^ como capaz de produzir analgesia à pressão, tanto em alta (100Hz) quanto em baixa frequência (4Hz) na máxima intensidade tolerável. Um cuidado realizado no presente estudo foi a padronização da temperatura inicial, previamente à primeira avaliação. Em estudo feito por Morimoto et al.,^(8)^ antes de cada avaliação, o membro avaliado era imerso em água morna, para a temperatura se apresentar uniforme em cada uma das avaliações. Tal cuidado não dispensado no presente estudo pode ter causado alteração nos limiares de dor detectados.

Desse modo, salientam-se como limitações do estudo, além da diferença de sexo, a não estabilização de temperatura anterior a cada avaliação e a carência de um do grupo controle absoluto, ou seja, indivíduos que não tivessem passado por nenhum tipo de estimulação elétrica, o que não ocorreu aqui por se tratar de estudo cruzado com todos os voluntários recebendo todas as formas de eletroestimulação.

Para futuros estudos, sugere-se atenção para tais questões, além de protocolos com tempo de tratamento superior a 20 minutos e, principalmente, o uso de intensidades maiores próximas ao umbral de dor.

## CONCLUSÃO

O uso da estimulação elétrica nervosa transcutânea sobre os dermátomos de C6 a C8, com aplicação de 0, 7, 100 e 255Hz, não produziu alteração significativa no limiar de dor à pressão e nem no desconforto ao frio.
